# The effect of multiple outgrowths from bronchial tissue explants on progenitor/stem cell number in primary bronchial epithelial cell cultures from smokers and patients with COPD

**DOI:** 10.3389/fmed.2023.1118715

**Published:** 2023-10-13

**Authors:** Nuray Bostancieri, Kemal Bakir, Seval Kul, Ayhan Eralp, Ozgecan Kayalar, Nur Konyalilar, Hadi Rajabi, Mehmet Yuncu, Ali Önder Yildirim, Hasan Bayram

**Affiliations:** ^1^Department of Histology and Embryology, School of Medicine, University of Gaziantep, Gaziantep, Türkiye; ^2^Cell Culture Laboratory, Department of Chest Diseases, School of Medicine, University of Gaziantep, Gaziantep, Türkiye; ^3^Department of Pathology, School of Medicine, University of Gaziantep, Gaziantep, Türkiye; ^4^Department of Biostatistics, School of Medicine, University of Gaziantep, Gaziantep, Türkiye; ^5^Koc University Research Center for Translational Medicine, Koc University, Istanbul, Türkiye; ^6^Comprehensive Pneumology Center (CPC), Institute of Lung Health and Immunity (LHI), Member of the German Center for Lung Research (DZL), Helmholtz Munich, Munich, Germany

**Keywords:** COPD, smoker, progenitor/stem cell, primary bronchial epithelial cell culture, cytokeratin 5, cytokeratin 14, p63

## Abstract

**Background:**

Although studies suggest a deficiency in stem cell numbers in chronic airway diseases such as chronic obstructive pulmonary disease (COPD), the role of bronchial epithelial progenitor/stem (P/S) cells is not clear. The objectives of this study were to investigate expression of progenitor/stem (P/S) cell markers, cytokeratin (CK) 5, CK14 and p63 in bronchial epithelial explants and cell cultures obtained from smokers with and without COPD following multiple outgrowths, and to study this effect on bronchial epithelial cell (BEC) proliferation.

**Methods:**

Bronchial epithelial explants were dissected from lung explants and cultured on coverslips. Confluent cultures were obtained after 3–4 weeks’ (transfer, Tr1), explants were then transferred and cultured for a second (Tr2) and third (Tr3) time, respectively. At each stage, expression of CK5, CK14 and p63 in explants and BEC were determined by immunostaining. In parallel experiments, outgrowing cells from explants were counted after 4wks, and explants subsequently transferred to obtain new cultures for a further 3 times.

**Results:**

As the transfer number advanced, CK5, CK14 and p63 expression was decreased in both explants and BEC from both smokers without COPD and patients with COPD, with a more pronounced decrease in BEC numbers in the COPD group. Total cell numbers cultured from explants were decreased with advancing outgrowth number in both groups. Smoking status and lung function parameters were correlated with reduced P/S marker expression and cell numbers.

**Conclusion:**

Our findings suggest that the number of P/S cells in airway epithelium may play a role in the pathogenesis of COPD, as well as a role in the proliferation of airway epithelial cells, *in vitro*.

## Introduction

Progenitor/Stem (P/S) cells, which are not fully differentiated, may differ from other cells in their ability to proliferate over long periods of time and to regenerate themselves (1, 2). These cells contribute to the regeneration of local tissue when transferred to a damaged area (3). P/S cells also serve as a source for differentiated cells where there is no tissue damage present. The internal signals that induce this differentiation are mediated by genes within the cell, whereas external signals are mediated by physical contact with neighboring cells, chemicals and molecules present in the microenvironment (1, 2).

Even though the term “progenitor cell” is commonly used as a synonym for stem cell in the literature, this phrase also refers to oligopotent cells, and thus “progenitor cell” is used to describe cells standing between stem cells and fully differentiated cells (4). However, when referring to cells of pulmonary origin the terms, “progenitor” and “stem” cell are used together as “Progenitor/Stem cell” (5, 6).

The epithelial P/S cells of the respiratory tract originate from ductal cells in the sub-mucosal glands of the proximal trachea (these glands were only demonstrated in mice), from basal cells in the lower parts of the trachea and in the inter-cartilaginous zones of the bronchi, from variant club cells in the bronchiolar and Bronchio-Alveolar channel junction and from type 2 alveolar-epithelial cells (7). The main function of P/S cells in the epithelium of the respiratory tract is to provide airway homeostasis and to repair defects in the airway wall (8, 9).

P/S cells have been isolated through various techniques, and keratin proteins are commonly used as markers for identifying epithelial cells and distinguishing between the pseudo-stratified epithelium and the functional sub-groups of stratified epithelium (10, 11). Whether they are located in the glandular ductal epithelium, in the secretory or stratified squamous epithelium, all basal cells express cytokeratin (CK) 5 and CK14. In monitoring the distribution of basal cells, CK5 and CK14 expression was detected in basal cells of both the stratified epithelium and the mixed epithelial glands (12).

Moreover, p63 protein, which is structurally and functionally very similar to p53 (13), has important functions in the development of squamous epithelium, epidermal transformation, and in stem cell maintenance (14). p63 is expressed at higher levels in stem or progenitor cell populations, and in various epithelial tissues (15).

Although an intense airway and parenchymal inflammation has been observed in COPD, the mechanisms underlying tissue and cellular injury, and subsequent regeneration are not fully understood. Recent studies suggest that peripheral hematopoietic progenitor cells and circulating CD34, CD133 positive endothelial progenitor cells may play a role in COPD (16, 17). More recently, it has been reported that basal progenitor cell counts were reduced in airway epithelial cells of COPD patients (18).

Primary epithelial cultures from the airways are intensively used in respiratory research. P/S cell markers such as CK5, CK14 and p63, have been demonstrated in primary airway epithelial cultures immortalized for obtaining cell lines, and were shown to maintain expression of these markers as passage number increased (19). However, it is not clear whether the expression of these markers has a role in the proliferation and maintenance of primary human bronchial epithelial cells (BEC) obtained from lung explants.

We hypothesized that the number of P/S cells may be decreased in the bronchial epithelium of patients with COPD, and this may be maintained during culture conditions, which could lead to decreased ability of explants to generate cells, *in vitro*. The objectives of our study were; (i) to investigate the expression of P/S cell markers CK5, CK14 and p63 in bronchial explants and BEC cultures outgrown from explants obtained from smokers without COPD and patients with COPD, (ii) to examine explants and cultures for changes in the expression of P/S markers as the number of outgrowths increased, (iii) to investigate the effect this has on the number of BECs outgrown from explants.

## Materials and methods

### Study patients

**Figure 1:**
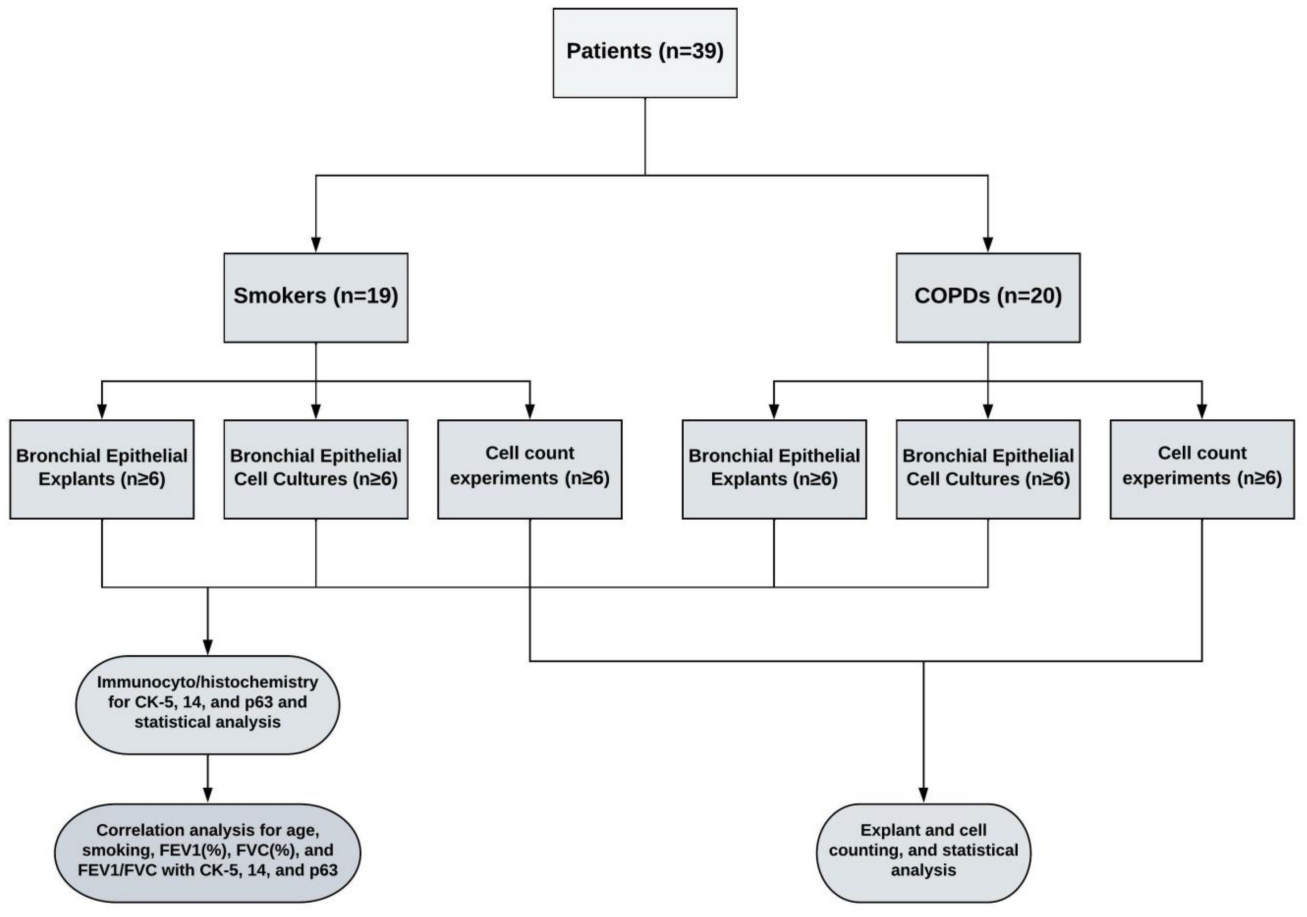
The study design.

Lung explants were obtained from 20 patients (all males), who were smokers with COPD and had COPD at stage 1 or above according to the guidelines for the global initiative for obstructive lung disease (GOLD) (20), and from 19 patients (2 females, 17 males), who were smokers but had no obstructive pulmonary disease. Smoker is defined by the Centre for Disease Control and Prevention (CDC) as “an adult who has smoked 100 cigarettes in his or her lifetime” (21). However, we included smokers, who consumed at least 1 pack/year cigarettes in their lifetime. The mean age of COPD patients (64.3 ± 1.6 years) was higher than the age of smokers (54.3 ± 2.6 years, *p* < 0.01). The smoking status as pack/years was more severe in COPDs (73.0 ± 7.5 pack/years) as compared to smokers (40.7 ± 6.3 pack/years, *p* < 0.01). Although forced expiratory volume in 1st second (FEV_1_) and forced vital capacity (FVC) percentages were similar in both groups, the rate of FEV_1_/FVC was significantly lower in COPD patients (62.6 ± 1.34%, *p* < 0.0001) compared to smokers (76.4 ± 1.9%) (Table 1). The study design is presented in Figure 1.

**Table 1:** Demographics and clinical characteristics of the study subjects.

	Smokers	COPD
Subjects (n)	19	20
Mean age years	54.26 ± 2.59	64.25 ± 1.62*
Smoking (packs/year)	40.68 ± 6.26	73.00 ± 7.47*
FEV1 (%)	86.37 ± 3.97	82.95 ± 4.87
FVC (%)	96.00 ± 5.31	101.5 ± 4.89
FEV1/FVC (%)	76.42 ± 1.91	62.60 ± 1.28**

Smokers, smokers without COPD; COPD, chronic obstructive pulmonary disease; FEV1, forced expiratory volume in the first second; FVC, forced vital capacity. *p < 0.01, **p < 0.0001 vs smokers without COPD.

Before entering the study, the patients were clinically stable and had received no systemic antibiotics, steroids, cytostatic medications, or radiotherapy. Patients were administered short-acting beta-2 agonists or anti-cholinergic treatments, as needed. None of the study subjects had upper or lower airway infections within the first month of the study. The study was approved by The Ethics Committee of Gaziantep University (Ref no: 01/2011–08), and informed written consents were taken from study volunteers.

### Reagents

All the reagents used for cell culture, if not stated otherwise, were supplied by the Sigma Chemical Company (Interlab, Turkey).

### Bronchial tissue

Lung explants from patients, who had lobectomy or pneumonectomy for lung cancer at Gaziantep University, Şahinbey Research and Training Hospital, were used to obtain primary cell cultures. The tissue confirmed as “normal” and macroscopically tumor free by a pathologist was placed in cold transfer medium (Medium 199 containing 0.5 mL/mL gentamycin) and transferred to the cell culture laboratory. The explant was processed for cell culture work within 0.5–1 h of resection.

### Isolation, culture, and identification of primary bronchial epithelial cells

The primary BEC cultures were obtained by an explant cell culture technique developed by Devalia et al. and described in detail previously (22, 23). The bronchial explant was examined under a dissection microscope, and the epithelial layer was dissected from the underlying tissue. The epithelium was cut into 1–2 mm^3^ pieces in size, and all explants were washed by pre-warmed and pre-gassed medium 199 containing 1% (vol/vol) antibiotics/antimycotic solution three times. Consequently, adequate numbers of explants were placed on coverslips, which were placed in 6-well culture plates or into cell culture dishes in cell culture medium 199 containing fetal calf serum (FCS), bovine pancreatic insulin, human transferrin, hydrocortisone, and antibiotic/antimycotic solution for P/S cell analysis or cell counting studies, respectively (22). These were incubated at 37°C in 5% CO_2_ in air atmosphere, and medium was replaced with culture medium containing Nu-serum IV (Becton Dickinson Biosciences, Turkey) after 3 days and then every 48 h for 3 to 4 weeks until the outgrowing cells had grown to confluence. The identity and purity of cells were checked in randomly selected cultures and confirmed by indirect immunoperoxidase staining techniques and light microscopy, as described before (22, 24). Prior to experimentation, bronchial explants were removed, and all cultures were incubated for 24 h in serum-free medium (SF) containing medium-199 and antibiotics/antimycotic solution for equilibration of cultures. Consequently, the cultures were gently washed 3 times with fresh, pre-warmed and pre-gassed SF medium, and sets of at least 6 different cultures (each from a different subject) obtained from smokers and patients with COPD, were used for P/S cell studies and cell counting experiments. However, it was not always possible to obtain sufficient amount of bronchial epithelial explant from each patient donor to establish enough cultures for all designed experiments; therefore, not every experimental group had to contain BEC cultures from all patients.

### Effect of outgrowth number on P/S cell expression in bronchial explants and primary bronchial epithelial cell cultures

In this group of experiments, the change in the number of P/S cells in the bronchial epithelial explants used for primary culture was determined by expression of CK5, CK14 and p63 markers. At each stage, the cell cultures outgrown from bronchial explants were stained for the expression of CK5, CK14 and p63. At the beginning, 2–3 explants that were not used for culture were processed using a short tissue follow-up program, and a paraffin blocking protocol. These explants were regarded as being at transfer (Tr) 1. Other tissue explants were placed on coverslips (2 explants on each of coverslips with 22 × 22mm in size), which were placed into 6-well (3 cm in diameter)-BD Falcon™ culture plates (Becton Dickinson) containing FCS medium, and incubated and treated, as described above. At the end of the first 3–4-week period, some of the explants used for cell cultures were collected and regarded as Tr2 and underwent the same tissue follow-up and paraffin blocking procedure. Cell cultures outgrown on coverslips were regarded as Tr1 and stained for P/S markers. The remaining explants were transferred onto new coverslips in culture plates and were treated for another 3–4 weeks’ period in culture. At the end of this culture period, half of explants were removed and processed for immune staining (Tr3). Cell cultures were also immune-stained and regarded as Tr2. The rest of explants were transferred onto coverslips to obtain cell cultures for Tr3, and at the end of culture period. The cells that were outgrown from explants at Tr1, Tr2, and Tr3 were immune stained for P/S cell markers. The study design is outlined in Figure 1.

### Immune staining of bronchial epithelial tissue explants

Firstly, the blocked explant was cut into 5 μm thick pieces using a Leica RM 2245 microtome (Leica, Germany) and placed on positively charged slides and deparaffinized. Slides to be stained with anti-CK5 (Cat. no: NCL-L-CK5, Novocastra™, Newcastle, United Kingdom), and CK14 (Cat. no: NCL-L-LL002, Novocastra™) antibodies were placed in 10% citrate buffer solution, whereas those stained for p63 (Cat. no: NCL-L-p63, Novocastra™) were placed into a buffer solution containing 4% ethylenediaminetetracetic acid (EDTA) and boiled twice for 10 min. The slides were kept in 3% of H_2_O_2_ for 10 min. Consequently, they were incubated with antibodies directed toward CK5 (1:100), CK14 (1:20), and p63 (1:25) at room temperature for 60 min. After the washing with PBS, slides were incubated with a biotinylated secondary antibody (Goat anti-polyvalent, Thermo Scientific, United States), followed by horseradish-peroxidase (HRP) streptavidin treatment and 3,3′- diaminobenzidine tetrahydrochloride (DAB) chromogenous staining. During each staining procedure, a PBS-treated slide, and a slide containing bronchial tissue, were used as negative and positive controls, respectively.

### Immune staining of bronchial epithelial cell cultures

Cell cultures grown on coverslips were washed with phosphate buffered saline (PBS), and then fixed using 4% paraformaldehyde. Following H_2_O_2_ treatment, cells were permeabilized with tween-containing PBS (PBS-T) at + 4°C. A serum block was performed at room temperature, and cultures were then incubated with anti-CK5, CK14 and p63 at the same dilutions as used for the paraffin sections. The remaining procedure was the same as the protocol used for tissue explants explained above.

### Analysis of tissue sections and cell culture slips following immune staining

After the staining process, immune-stained cells were counted under a light microscope with the x40 objective in 10 randomly chosen different fields for each preparation by two different histologists, who were blinded to the diagnosis of tissue donors. The average of cells counted in these fields was used for statistical analysis.

### The effect of outgrowth number on the number of cells obtained from bronchial explants

In these studies, we investigated effects of outgrowth number on cell-generating capacity of bronchial epithelial explants under *in vitro* culture conditions. An average of 6 bronchial explants from each donor with 1–2 mm^3^ in size were placed into 10 cm-BD Falcon™ culture plates (Becton Dickinson) containing culture medium, and they were observed for cell outgrowth, as described above. During the culture period, explants, which were not attached to culture plates, were discarded. At the end of the 3–4 weeks’ period, explants were transferred to new culture plates, and cells outgrown from explants were counted under a light microscope using “trypan blue” dye. The total cell counts in each plate and cell numbers per explants were noted. The same procedure was repeated at the end of the second (Tr2) and third (Tr3) 3–4 weeks’ period.

### Statistical analysis

Data were tested for normality, and continuous variables were compared using one-way variance analysis, ANOVA / Dunnett’s multiple comparison tests or the Kruskal-Wallis/ Dunn’s multiple comparison tests. Unpaired t test or Mann–Whitney U tests were used to compare smokers and COPD subjects. Generalized estimating equations were used to evaluate changes in P/S marker expressions, and number of cells generated by bronchial explants over repeated measurements, and to determine effect of possible related variables such as age, cigarette smoke (packs/year), FEV1 and FVC on dependent variables. Adjusted odds ratios (OR) and 95% confidence intervals (CI) were given to show the effect of independent variables. Spearman rank correlation was performed to evaluate correlations between numerical variables, and the correlation coefficients (“*r*”) were presented. Results are expressed as mean ± SEM or median ± interquartile ranges (Q1 and Q3). *p* values smaller than 0.05 were regarded as significant. Statistical analysis was performed using PRISM version 6 (GraphPad Software Inc, San Diego, CA, United States) and SPSS for windows version 22.

## Results

### Primary bronchial epithelial cell culture

**Figure 2:**
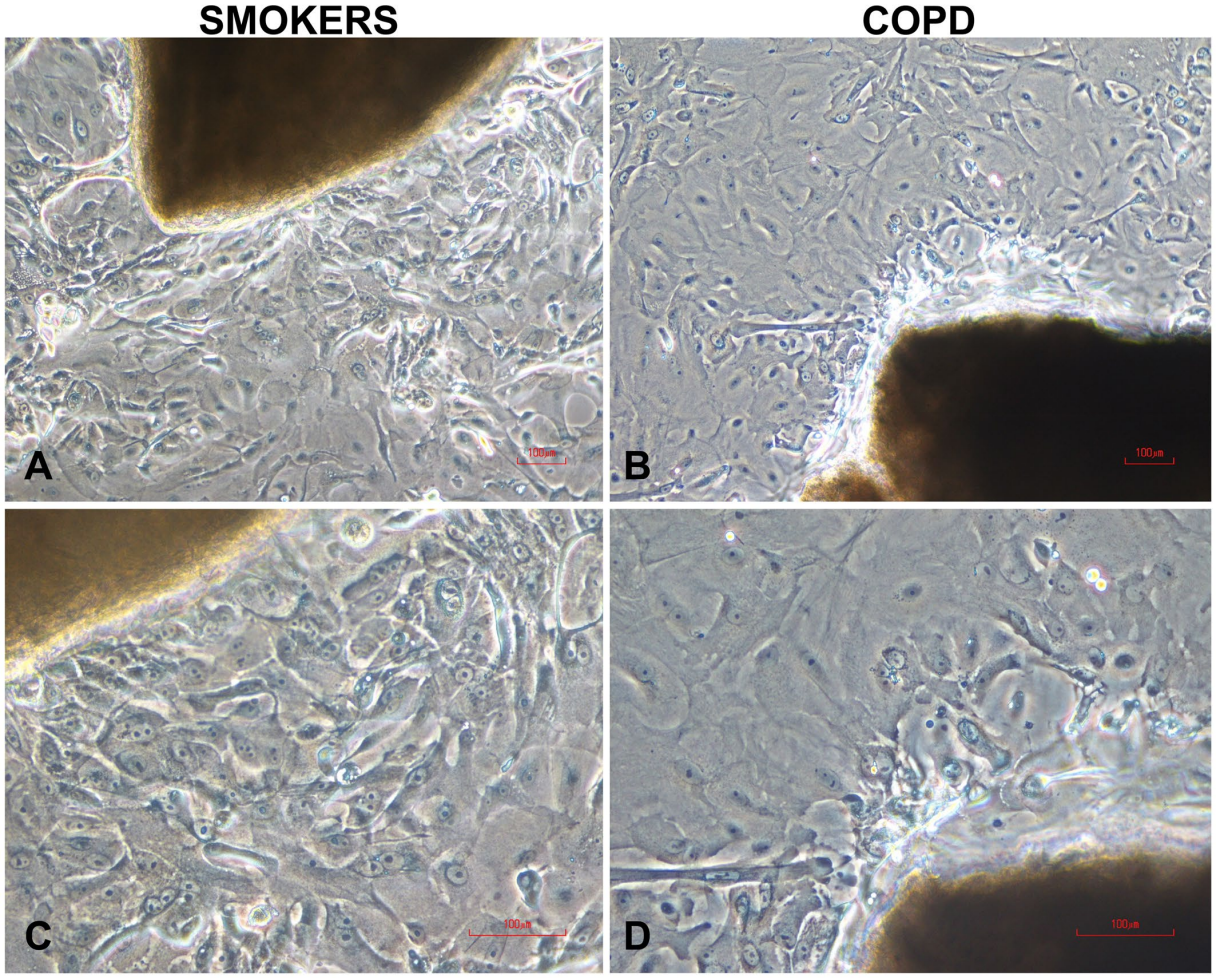
Typical human bronchial epithelial explants and outgrowing epithelial cells in two-weeks culture from smokers without COPD **(A,C)** and COPDs **(B,D)**. Magnifications: x10 for **(A,B)**; x20 for **(C,D)**; the scale bars: 100 μm.

Cells cultured on coverslips for P/S experiments started to proliferate from day 7 onwards, and by the end of the 3rd week, they sufficiently covered the surface of the coverslips. In experiments, where we investigated the effects of multiple outgrowths on the number of cells obtained from bronchial explants, bronchial epithelial cells started to outgrow from explants at days 4 or 5 onwards, and there was adequate cell proliferation by the end of the 3rd week. Figure 2 shows outgrowing BECs from the explant of both a smoker subject (Figures 2A,C) and a patient with COPD (Figures 2B,D) under the light microscope.

### The expression of P/S markers in bronchial epithelial explants, and the effect of multiple outgrowths

**Figure 3:**
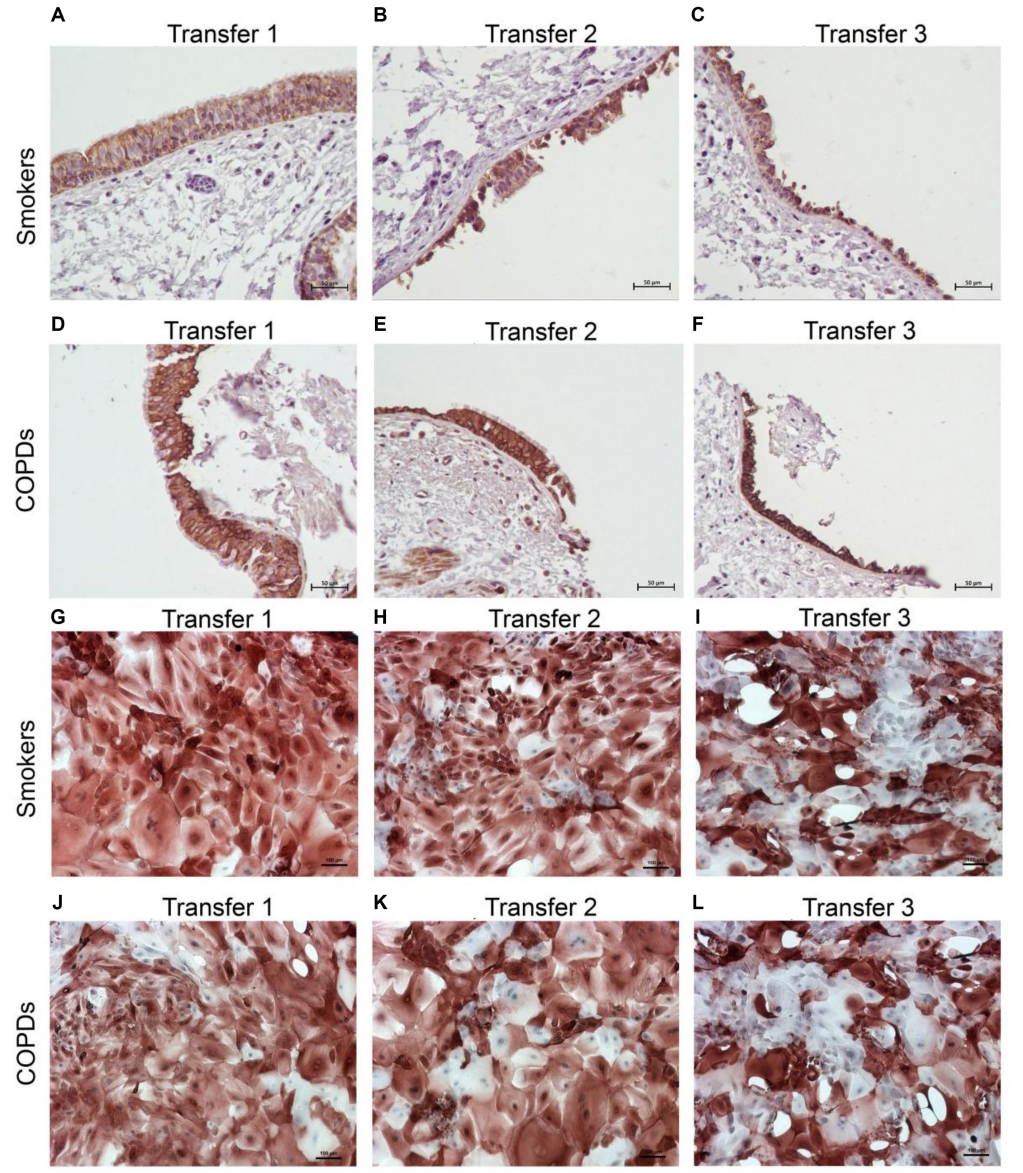
Immunostaining of bronchial epithelial cell explants and cultures for cytokeratin (CK) 5. Explants: (i) Smokers without COPD: **(A)** Transfer 1; **(B)** transfer 2; **(C)** transfer 3, (ii) COPDs: **(D)** transfer 1; **(E)** transfer 2; **(F)** transfer 3. Cultures: (i) Smokers without COPD: **(G)** transfer 1; **(H)** transfer 2; **(I)** transfer 3, (ii) COPDs: **(J)** transfer 1; **(K)** transfer 2; **(L)** transfer 3. The scale bar = 50 μm. Brown color indicates positive immunoreactivity for CK5 in cytoplasm of the cells.

**Figure 4:**
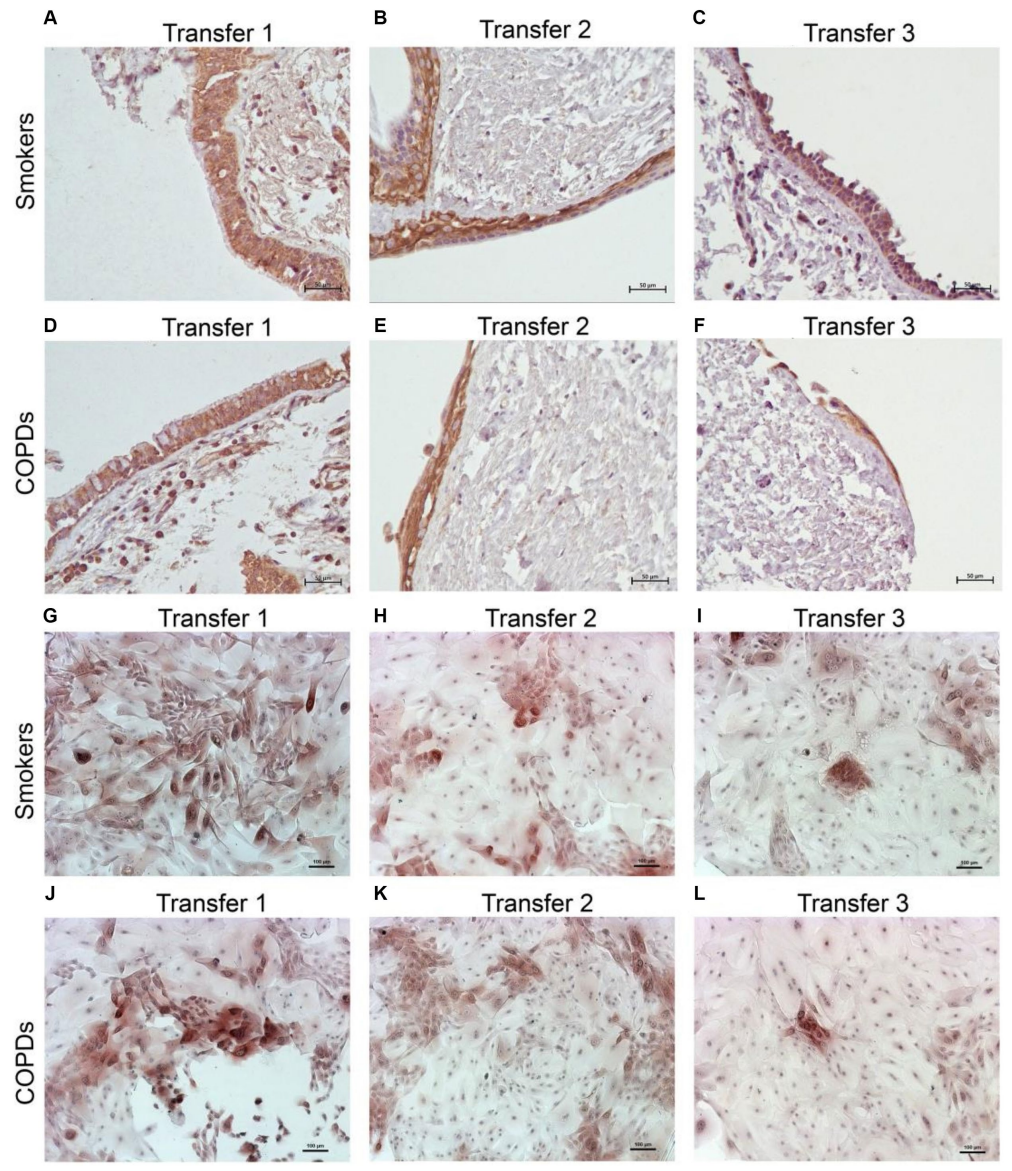
Immunostaining of bronchial epithelial cell explants and cultures for cytokeratin (CK) 14. Explants: (i) Smokers without COPD: **(A)** transfer 1; **(B)** transfer 2; **(C)** transfer 3, (ii) COPDs: **(D)** transfer 1; **(E)** transfer 2; **(F)** transfer 3. Cultures: (i) Smokers without COPD: **(G)** transfer 1; **(H)** transfer 2; **(I)** transfer 3, (ii) COPDs: **(J)** transfer 1; **(K)** transfer 2; **(L)** transfer 3. The scale bar = 50 μm. Brown color indicates positive immunoreactivity for CK14 in cytoplasm of the cells.

**Figure 5:**
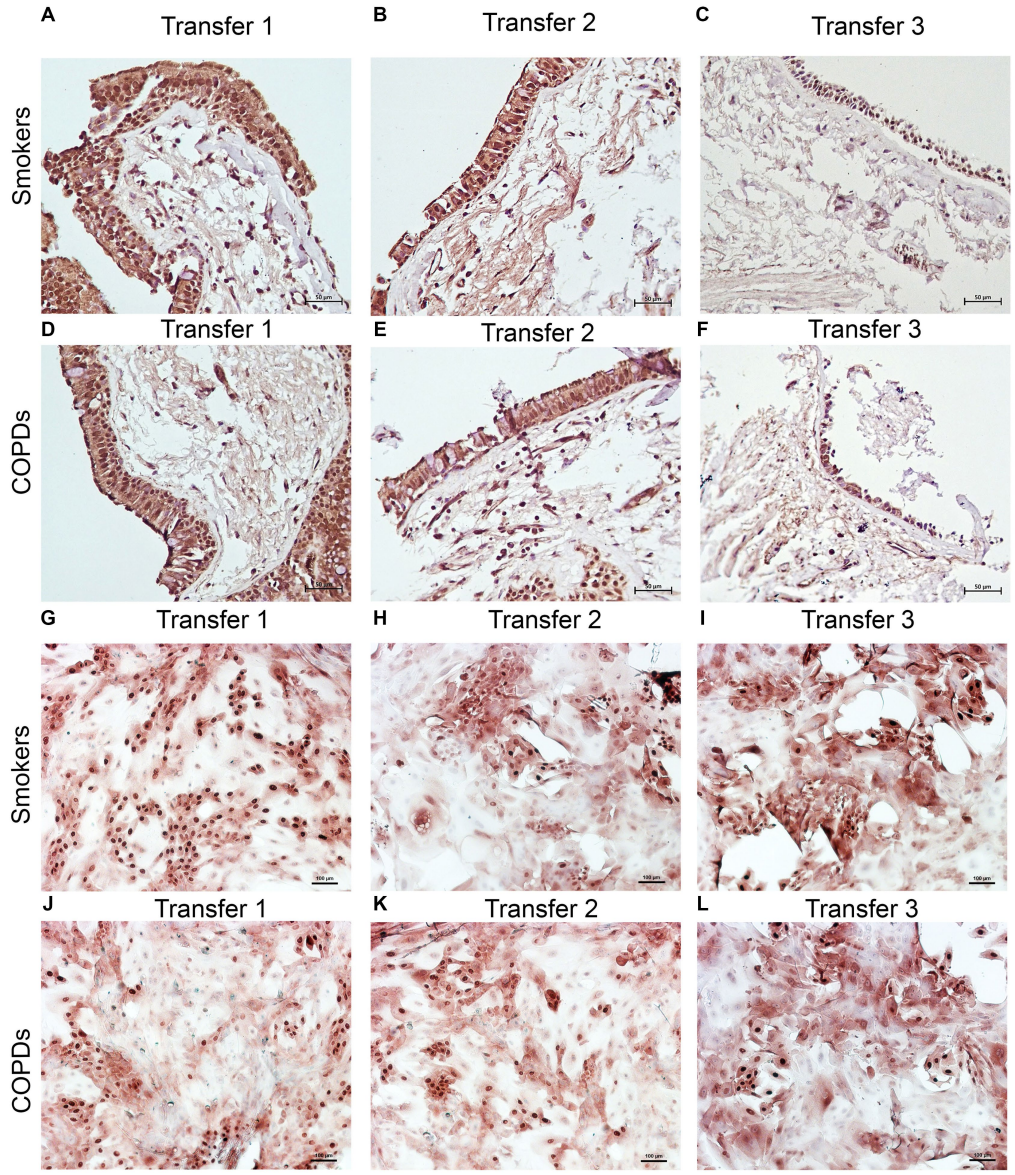
Immunostaining of bronchial epithelial cell explants and cultures for p63. Explants: (i) Smokers without COPD: **(A)** transfer 1; **(B)** transfer 2; **(C)** transfer 3, (ii) COPDs: **(D)** transfer 1; **(E)** transfer 2; **(F)** transfer 3. Cultures: (i) Smokers without COPD: **(G)** transfer 1; **(H)** transfer 2; **(I)** transfer 3, (ii) COPDs: **(J)** transfer 1; **(K)** transfer 2; **(L)** transfer 3. The scale bar = 50 μm. Brown color indicates positive immunoreactivity for p63 in nuclei of the cells.

**Figure 6:**
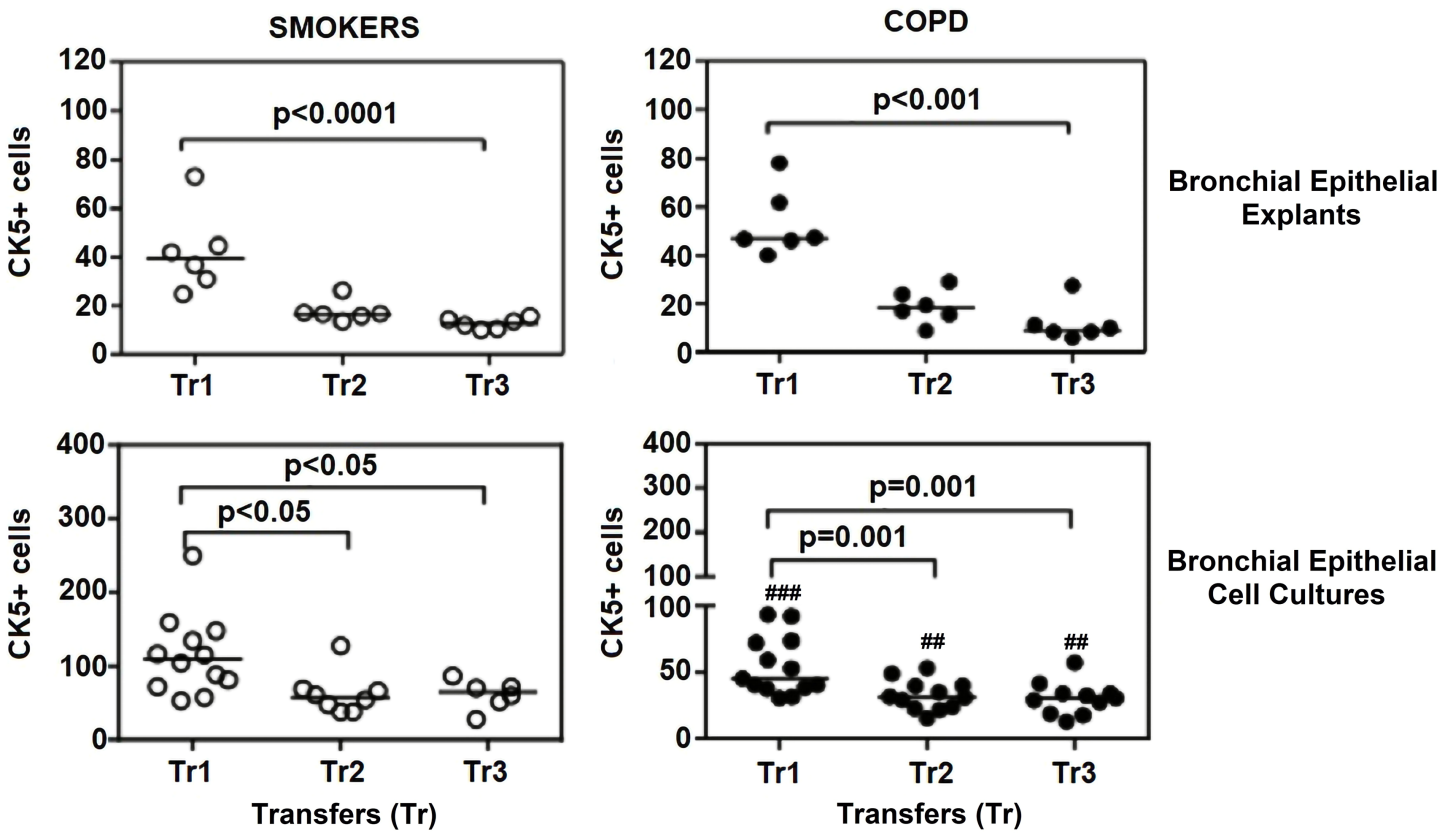
Effect of outgrowth number of transfers (tr) on number of cells expressing cytokeratin (CK) 5 in bronchial epithelial explants, and bronchial epithelial cell cultures of smokers without COPD and COPDs (##*p* < 0.01 and ###*p* < 0.001 vs. smokers).

**Figure 7:**
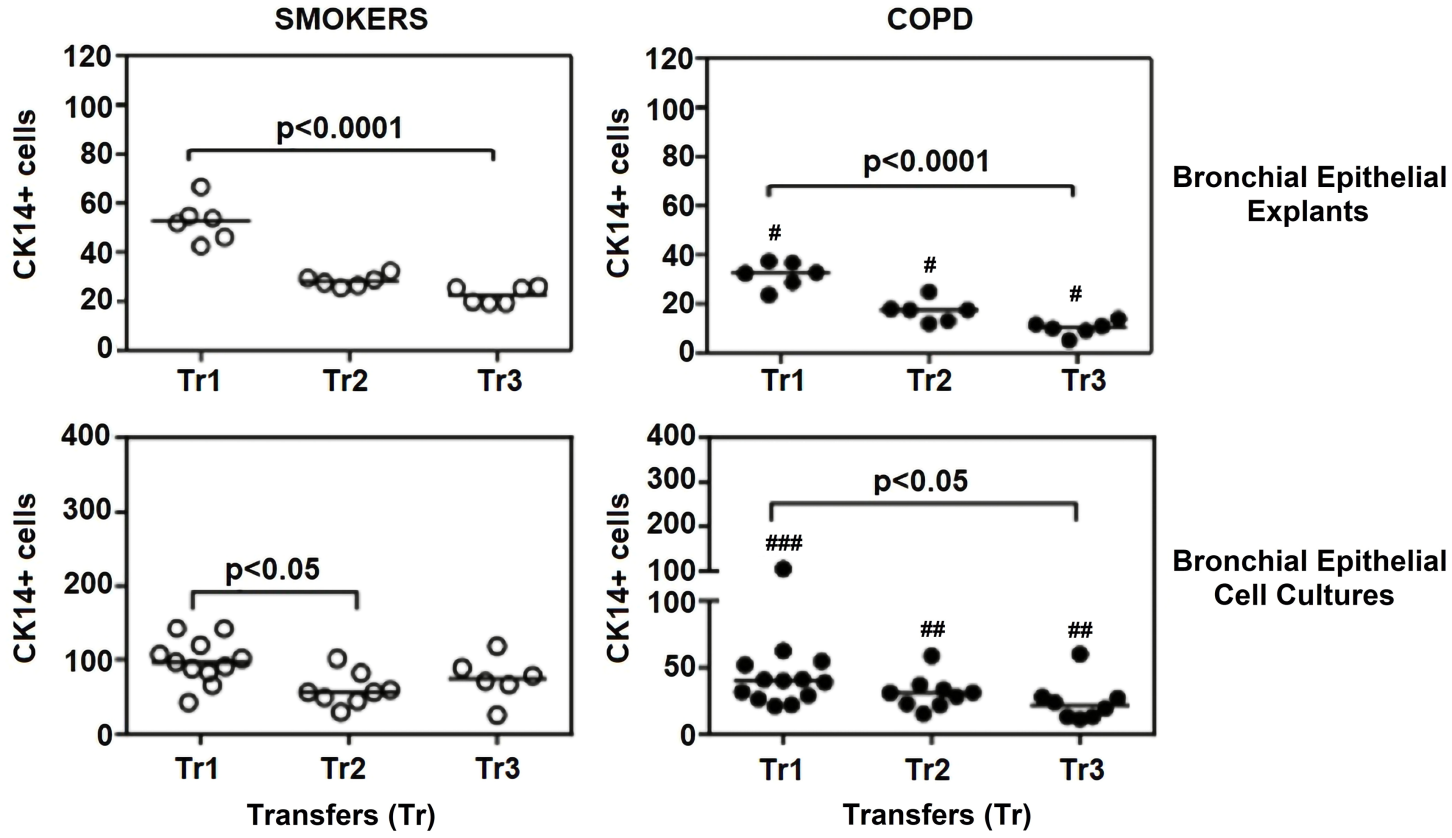
Effect of outgrowth number of transfers (tr) on number of cells expressing cytokeratin (CK) 14 in bronchial epithelial explants and bronchial epithelial cell cultures of smokers without COPD and patients with COPD. ##*p* < 0.01 and ###*p* < 0.001 vs. smokers without COPD.

**Figure 8:**
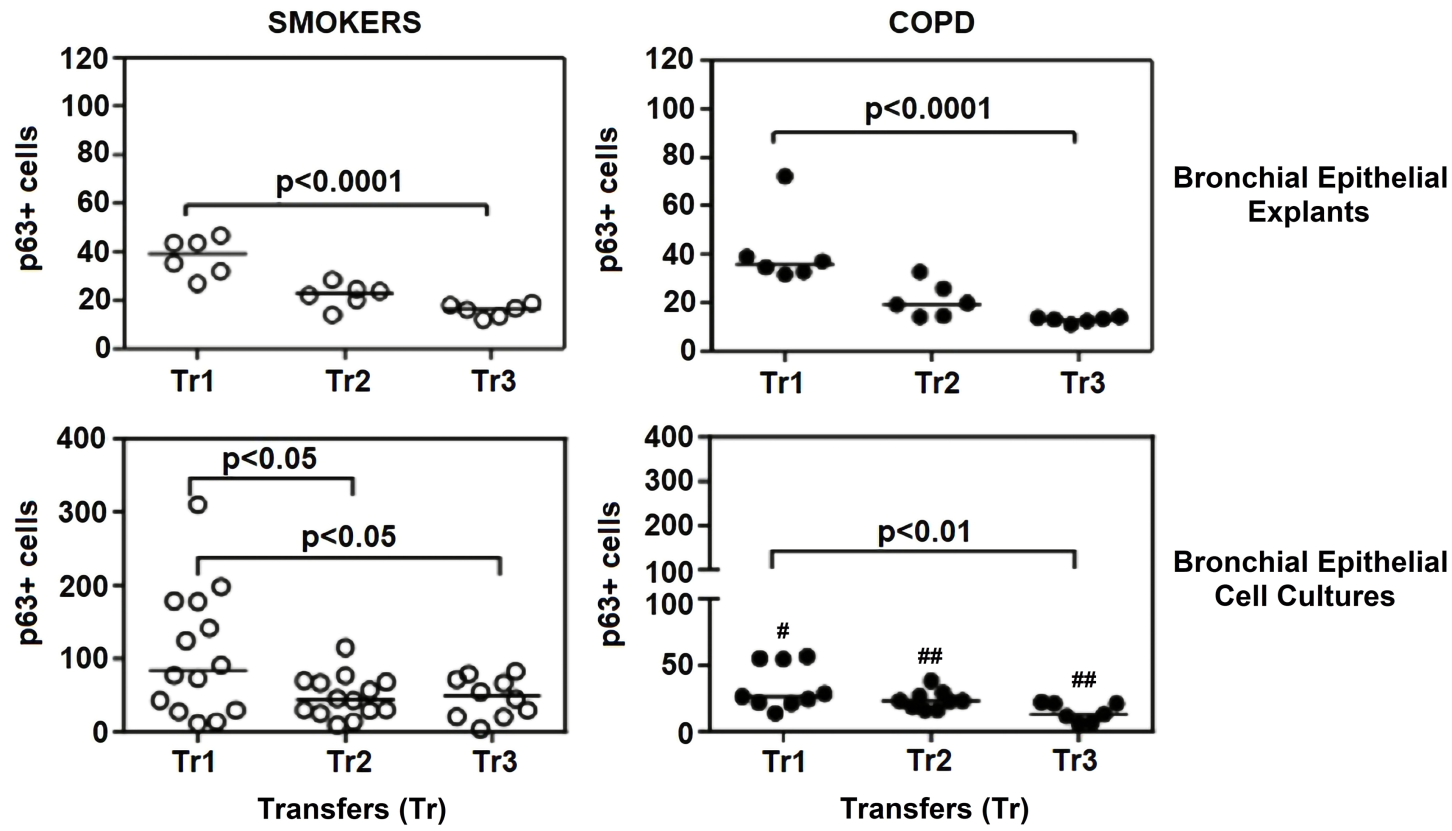
Effect of outgrowth number of transfers (tr) on number of cells expressing p63 in bronchial epithelial explants and bronchial epithelial cells cultured from smokers without COPD and patients with COPD. #*p* < 0.05 and ##*p* < 0.01vs smokers without COPD.

**Figure 9:**
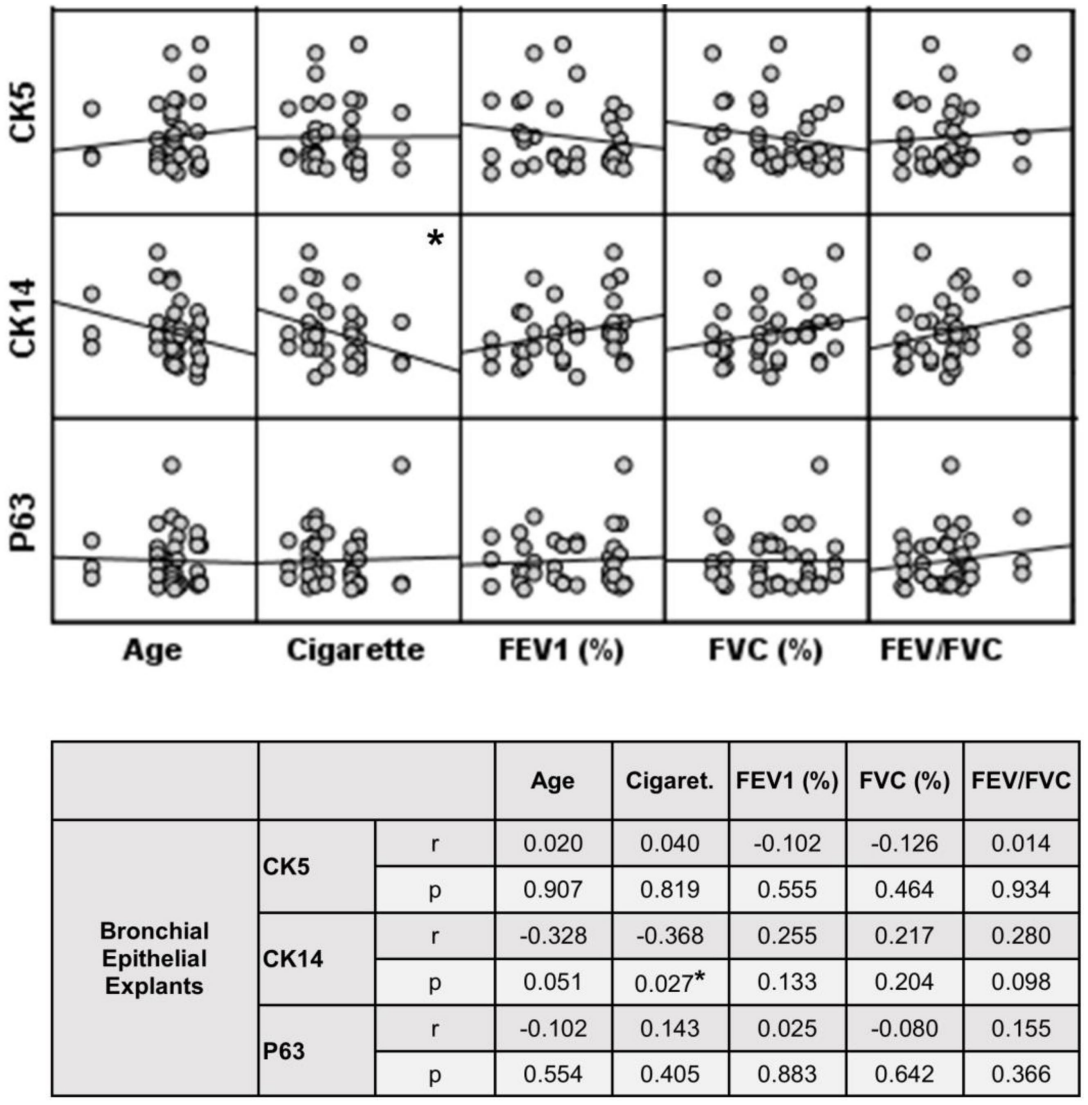
Correlation between age, smoking status as pack/years, lung function parameters (FEV1%, FVC%, and FEV/FVC) and expression of CK5, CK14 and p63 in bronchial epithelial explants. Correlation coefficients (*r*) and *p* values are presented in the table below the figure.

There was a gradual decrease in the expression of P/S markers with increased outgrowth number in explants from both smokers and COPD patients. Immunohistochemical staining of explants from both groups can be seen at Tr1-Tr3 in Figures 3–5. The expression of CK5, CK14, and p63 markers in the explants obtained from both COPDs and smokers, and the effects of multiple tissue transfers are summarized in Figures 6–8 and Supplementary Table S1. Furthermore, the correlation between outgrowth number, age, cigarette smoke, and lung function parameters and expression of CK5, CK14, and p63 in the explants are presented in Figure 9 and Supplementary Figure S1.

#### Cytokeratin 5

When the expression of CK5 in advanced transfers was compared to the initial values at Tr1, a clear decrease was observed in smokers, and there was a significant decrease in CK5 expressing cells at Tr1 and Tr2 (*p* < 0.0001; Figure 6). A similar trend was observed in explants from COPD patients, and the number of cells stained with CK5 antibody significantly decreased, as the outgrowth number increased (*p* < 0.001; Figure 6).

#### Cytokeratin 14

The expression of this marker significantly decreased with increasing Tr numbers in smokers, as was the case for CK5 in these subjects (*p* < 0.0001; Figure 7). Similarly, the expression of CK14 was significantly reduced in explants from COPD patients (*p* < 0.0001; Figure 7). The expression of CK14 in explants was weakly and negatively correlated with cigarette smoke (pack/years) (*r* = −0.368, *p* = 0.027) (Figure 9); however, when data of both groups of COPDs and smokers without COPD were separately analyzed, this became non-significant (Supplementary Figure S1).

#### p63

The expression of p63 in the bronchial epithelial explants also showed a significant decrease concomitant with increasing Tr number in both smokers without COPD (*p* < 0.0001; Figure 8), and patients with COPD (*p* < 0.0001; Figure 8).

When the P/S cell marker expression from the explants of both groups was compared, no significant difference was found in the expression of CK5 or p63 at Tr1, Tr2 or Tr3. However, CK14 expression was significantly decreased in COPD explants (*p* = 0.001) compared to explants from smokers at Tr1, Tr2 and Tr3 (Figure 7; Supplementary Table S1).

### The expression of P/S markers in bronchial epithelial cell cultures, and the effect of outgrowth number

Here, we investigated the presence of P/S markers by immunocytochemical staining for CK5, CK14 and p63 in primary BEC cultures (Figures 3–5). The change in marker expression was monitored in relation to outgrowth number (Figures 6–8; Supplementary Table S2).

#### Cytokeratin 5

**Figure 10:**
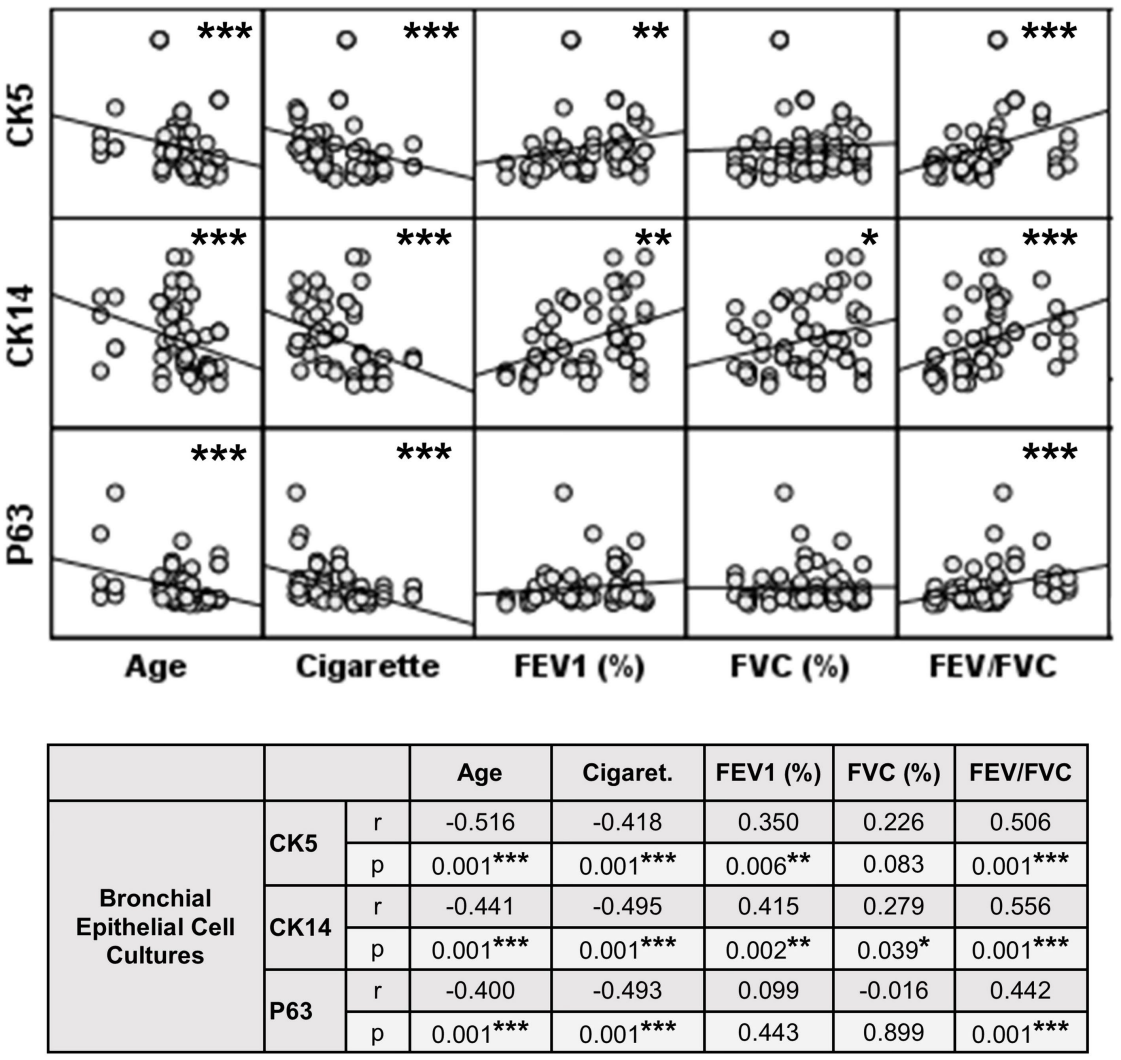
Correlation between age, smoking status as pack/years, lung function parameters (FEV1%, FVC%, and FEV/FVC) and expression of CK5, CK14 and p63 in bronchial epithelial cell cultures. Correlation coefficients (*r*) and *p* values are presented in the table below the figure.

The expression of this marker was significantly decreased at Tr2 and Tr3 in smokers as compared to Tr1 (*p* < 0.05, Figure 6). Similarly, CK5 expression was lower at Tr2 (*p* = 0.001) and Tr3 (*p* = 0.001) in COPD cultures than the expression observed at Tr1 (Figure 6). CK5 expression was negatively correlated with age (*r* = −0.516, *p* < 0.0001), and cigarette smoke (packs/year; *r* = −0.418, *p* = 0.001), whereas FEV1 (*r* = 0.35, *p* = 0.006) and FEV1/FVC *r* = 0.506, *p* < 0.0001) had a positive correlation with CK5 expression (Figure 10). As we analyzed the correlation between CK5 expression and age, cigarette smoke (pack/years) and lung function parameters in smokers with and without COPD separately, we found that the expression of this marker was negatively correlated with age (*r* = −0.334, *p* = 0.047), and positively associated with FVC (*r* = 0.335, *p* = 0.046) in only BEC cultures of COPD patients (Supplementary Figure S2).

#### Cytokeratin 14

When the expression of this marker was analyzed, the expression at Tr2 (*p* < 0.05) demonstrated a significant decrease in BEC cultures from smokers as compared to Tr1, whereas no significant difference was detected at Tr3 (Figure 7). On the other hand, CK14 expression was significantly reduced in BEC cultures obtained from COPD patients at Tr3 (*p* < 0.05) compared to Tr1, and there was no significant change at Tr2 (Figure 7). There was a negative correlation between CK14 expression and age (*r* = −0.441, *p* = 0.001), and smoking status (packs/year; *r* = −0.495, *p* < 0.001), in contrast FEV1 (*r* = 0.415, *p* = 0.002), FVC (*r* = 0.279, *p* = 0.039), and FEV1/FVC (*r* = 0.556, *p* < 0.0001) was positively correlated with CK14 expression (Figure 10). However, when data of both groups were separately analyzed, age (*r* = −0.382, *p* = 0.021) and cigarette smoke (pack/years) (*r* = −0.535, *p* = 0.001), and FVC (*r* = 0.353, *p* = 0.035) were significantly correlated with CK14 expression in only BEC cultures of COPDs (Supplementary Figure S2).

#### p63

Expression of p63 in BEC cultures from smokers showed a significant decline at both Tr2 and Tr3, as compared to Tr1 (*p* < 0.05, Figure 8). In COPD patients, however, a significant decrease in p63 expression was determined only at Tr3 (*p* < 0.01) compared to Tr1 (Figure 8). There was a negative association between p63 expression and age (*r* = −0.400, *p* = 0.001), and cigarette smoke (*r* = −0.493, *p* < 0.001). However, p63 was positively correlated with FEV/FVC (*r* = 0.442, *p* < 0.001) (Figure 10). As, the correlation analyses were performed in both groups separately, age (*r* = −0.572, *p* = 0.001) and cigarette smoke (pack/years) (*r* = −0.194, *p* = 0.001), were negatively, and FEV_1_ (*r* = 0.415, *p* = 0.016), and FVC (*r* = 0.446, *p* = 0.009) were positively correlated with expression of CK14 in BEC cultures of COPDs. In smokers without COPD, cigarette smoke (packs/year; *r* = −0.382, *p* = 0.028) was negatively correlated with CK14 expression, while FEV1/FVC (*r* = 0.405, *p* = 0.019) was positively correlated with the expression of this marker (Supplementary Figure S2).

A comparison of P/S cell markers between the two groups demonstrated that CK5, CK14, and p63 expressions at all outgrowths of Tr1, Tr2, and Tr3 were lower in BEC cultures from COPD patients than smokers (Figures 6–8; Supplementary Table S2).

### The effect of multiple outgrowths on cell counts from bronchial epithelial cell cultures

**Figure 11:**
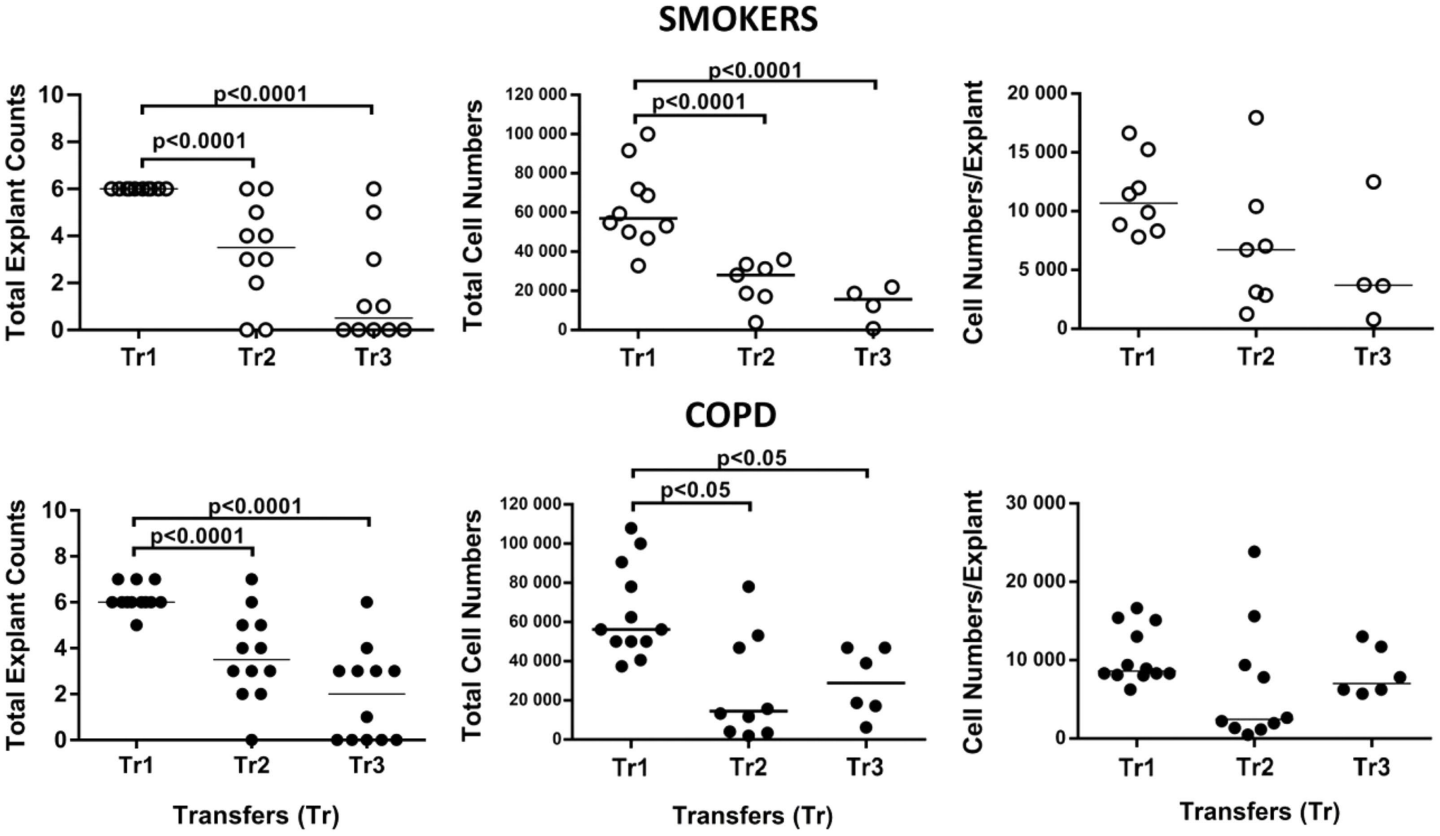
Effect of transfer (tr) numbers on number of explants adhering to culture dishes and producing cells in culture, total cell numbers produced by explants in culture, and number of cells per bronchial explant of smokers without COPD and COPDs.

At the beginning, an average of 6 explants were placed into culture plates from smokers, whereas 6.1 explants from patients with COPD were added to culture plates. However, all these explants did not attach to the plates and generate cells. Consequently, at the end of Tr1, the number of explants transferred to Tr2 and Tr3 from both smokers and COPD patients significantly decreased (*p* < 0.0001; Figure 11; Supplementary Table S3).

When the total number of cells were analyzed, the number of outgrowing cells from explants of both smokers (*p* < 0.0001) and COPD patients (*p* < 0.05) significantly decreased concomitant with increased Tr numbers, except total cell counts from the COPD group at Tr3 being higher than Tr2 (Figure 11; Supplementary Table S3).

**Figure 12:**
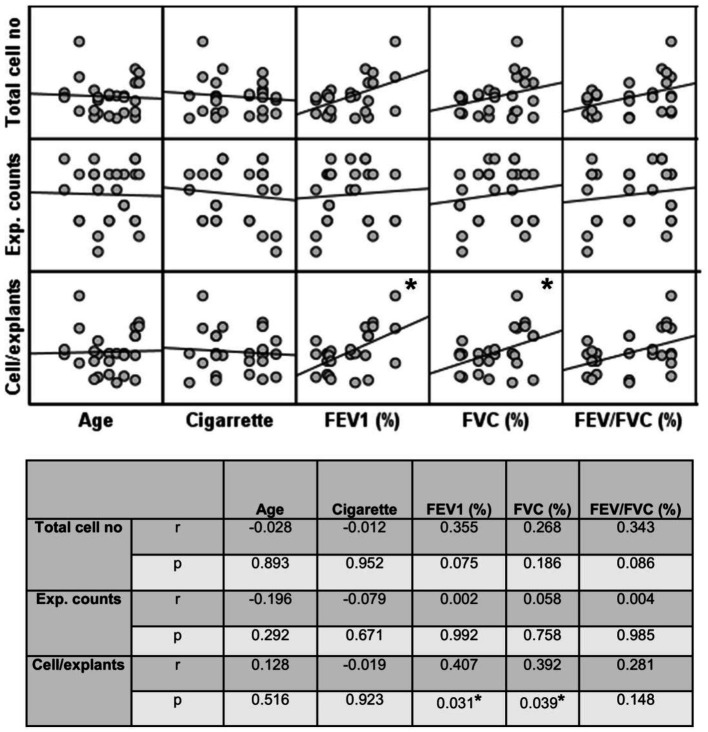
Correlation between age, smoking status as pack/years, lung function parameters (FEV1%, FVC%, and FEV/FVC) and number of explants generating cells, total epithelial cell counts, and cells/explants. Correlation coefficients (*r*) and p values are presented in the table below the figure.

Finally, the total number of cells was divided by the total number of explants used for culture. The change in the number of cells outgrown from each explant (cell numbers/explant) with increasing Tr number was therefore monitored. As the number of Trs increased, no significant difference was observed, even though the number of the cells per explant showed a tendency to decrease in both smokers and COPD patients (Figure 11; Supplementary Table S3). FEV1 (*r* = 0.407, *p* = 0.031), and FVC (*r* = 0.392, *p* = 0.039), had a positive correlation with the total number of cells generated by per bronchial epithelial explant (Figure 12). When data of both groups were separately analyzed, in smokers without COPD, cigarette smoke (pack/years) were positively correlated with total cell counts (r = 0.581, *p* = 0.003) and cell numbers from per explant (*r* = 0.707, *p* = 0.001), respectively. In contrast, FVC was inversely correlated with totals cell counts (*r* = −0.496, *p* = 0.014). In COPD group, FEV_1_ (*r* = 0.342, *p* = 0.033) and FVC (*r* = 0.360, *p* = 0.024) were correlated with cell counts from explants, while FEV_1_/FVC (*r* = 0.350, *p* = 0.046) was correlated with total cell counts (Supplementary Figure S3).

## Discussion

In the present study, we have investigated the presence of P/S cells in both bronchial explants and BEC cultures from smokers and patients with COPD. Furthermore, we studied the effects of multiple outgrowths of bronchial epithelial explants (tissue transfers) on the expression of P/S markers in both explants and BEC cultures, as well as the impact of this on the number of cells generated from bronchial explants. As the outgrowth number increased, the expression of P/S cell markers (CK5, CK14 and p63) in the BEC cultures and explants significantly decreased in both smokers and COPD patients, and this was most prominent in patients with COPD. In parallel with this, both the number of explants that generated cells, and the total number of cells obtained from these explants significantly decreased, as the number of outgrowths increased. Our results suggest that P/S cell expression in both BEC explants and cultures decrease with increased outgrowth number, and that P/S cells may play a role in the proliferation of airway epithelial cells, *in vitro*.

Although an intense airway and parenchymal inflammation has been observed in COPD, the mechanisms underlying the tissue and cellular injury and their regeneration are not adequately known. Studies suggest that P/S cells play a role in the pathogenesis of COPD. Indeed, it has recently been shown that basal progenitor cell counts were decreased in bronchial biopsies obtained from patients with COPD (18). Interestingly, the decrease in cell number correlated with disease severity.

In the present study, we found that the P/S cell marker CK14 was mildly lower in bronchial epithelial explants of patients with COPD than the control smoker group. Furthermore, the expression of P/S cell markers studied (CK5, CK14 and p63) was also decreased in BEC cultures from COPD patients, as compared to smokers. Our findings agree with the abovementioned studies reporting decreased levels of progenitor cells in bronchial biopsies (18). Indeed, studies have shown that P/S cells act as a source for other cells, playing a major role in tissue regeneration (8, 9). Taken together, these findings suggest that a possible decline in the number of the P/S cells might be responsible for the deficiency in repair of the damaged airway epithelium, as well as for the pathological changes observed to the airway epithelium in COPD (squamous metaplasia, increased goblet cell number and glands secreting mucus) (20, 25).

Moreover, we found that the expression of all P/S cell markers (CK5, CK14 and p63) decreased as the outgrowth number of explants increased in both smokers and patients with COPD. Although CK5 and p63 expression in explants from both groups did not show a significant difference, the decline observed with increased outgrowth number in CK14 expression (Tr1, Tr2) was greater in the explants with COPD than in those of smokers. In parallel with this, the expression of P/S markers in BEC cultures significantly decreased as the outgrowth number increased and decreases in COPD cells were more prominent compared to cells of smokers. No prior studies have examined P/S cell expression in explants or BEC cultures in advanced outgrowths from either non-COPD or COPD patients. These findings suggest that the capacity of BEC cultures in producing P/S cells was decreasing with advancing outgrowth number of explants.

Previous studies suggest that a dysfunction in the Yes-associated Protein 1 (YAP) and Transcriptional coactivator with PDZ-binding motif (TAZ) signaling pathway may play a role in the progenitor cell reduction in the airways in COPD. Developmental abnormalities such as abnormal alveolarization and airspace enlargement during lung development were observed in the lungs of TAZ knockout mice, leading to the emphysematous lung phenotype that is frequently observed in COPD (26, 27). Furthermore, the dysregulation of YAP/TAZ signaling pathway was reported to contribute to the development and progression of chronic lung diseases, including COPD and asthma (28). Indeed, Zhao et al. (29) reported that Yap, which interacts with p63 in basal stem cells, was required for the maintenance of airway basal stem cell identity. The overexpression of YAP increased stem cell proliferation resulting in epithelial hyperplasia, while YAP knockdown inhibited stem cell proliferation *in vitro*. The fact that the number of P/S cells expressing CK5+, CK14+ and p63+ were decreased in our BEC population with advancing outgrowth numbers, it is tempting to speculate that this might also be due to a dysfunction in the YAP/TAZ signaling pathway during this process.

On the other hand, the plasticity of airway epithelial cells has a key role in the maintenance of air way epithelium, and in response to a stimulus, epithelium can change cellular composition *via* activation of transcriptional programs that can lead to transdifferentiation of epithelial cell types (30). It has been reported that interleukin (IL)-4 stimulation can modulate cell proliferation, cilia beating and mucus production (31). Similarly, both IL-4 and interferon gamma (IFN-g) were shown to orchestrate epithelial polarization in the airways (32). It has been revealed that these cytokines, which are key regulators of type 1 (Th1) and type 2 (Th2) responses, may be related to the transcription factor network of airway epithelial cells (32). However, the effects of TH1/TH2 polarizing signals on airway epithelial cell plasticity have not been fully elucidated, yet. Therefore, it is important to directly investigate whether these key cytokines play a role in the proliferation or maintenance of P/S cells in airway epithelial cell cultures from healthy subjects or patients such as COPD in the future studies.

Our findings demonstrated that the decrease in P/S markers in both bronchial epithelial explants and BEC cultures was associated with decreased lung function parameters such as FEV_1_, suggesting that decreased lung function in COPD might have an impact on decreased P/S cell expression in airway epithelium. The impact of lung function parameters was found to be more prominent in BEC of COPDs, when the correlation analyses were separately performed. However, this effect was mild, and we think this was due to early stages of the disease in our study patients, since FEV_1_ and FVC values were similar in both smoker and COPD groups. These findings are still in agreement with those of others reporting decreased basal progenitor cells in bronchial biopsies of COPD patients (18).

Furthermore, cigarette smoke as pack/years had a negative effect on P/S expression in both explants and BEC cultures from both smokers without COPD and COPD patients. In our study population, smoking status was more severe in COPD, as compared to smokers. Thus, the more pronounced decrease in P/S markers in COPD than smokers might also be due to the increased tobacco consumption suggesting that cigarette smoke, as an independent risk factor, can suppress proliferation of P/S cells in airway epithelium. Similarly, increases in age were negatively associated with P/S cell marker expression in BEC cultures from smokers without COPD and COPD patients. However, this was more pronounced in COPDs. In line with our findings, studies report that stem cell exhaustion is an important feature of aging (33).

In parallel to the decrease in P/S cell expression with increasing outgrowth number, there was a significant decrease in the total number of cells obtained from the bronchial epithelial explants of both groups. Similarly, along with increasing outgrowth number, some of the explants, which adhered to culture plates and produced cells in earlier transfers, did not attach to culture plates to generate cells. Consequently, there was a decrease in the number of explants producing cells in both smokers and patients with COPD. The total cell counts, and cells produced by each explant were positively associated with FEV_1_ levels suggesting that better lung function has a positive impact on cell proliferation under *in vitro* culture conditions. This effect was consistent, even when the groups of smokers without COPD and COPD patients were separately analyzed. Similarly, smoking was positively associated with total cell numbers, particularly in smokers without COPD. Indeed, the earliest abnormality in the lung associated with smoking was reported as hyperplasia of airway epithelial cells (34).

However, there was no significant change in the number of the cells per explant along with increasing outgrowth number. This suggests that the decrease in total cell numbers generated by explants with advanced outgrowth number was due to the low adherence of explants to culture plates rather than decreases in their cell producing capacities. Nevertheless, we think more detailed studies are required in order to draw a firm conclusion. Conversely, it is possible that the low adherence of explants was due to their reduced capacity in generating cells in culture.

Our current findings seem to support the fact that there is a decline in both the number of BEC obtained from bronchial explants and the adherence of these explants to culture plates as the number of outgrowths increases, as we observed in our previous studies with asthmatic BEC cultures (35). Our findings also demonstrate that bronchial explants can be utilized for a limited time to obtain BEC cultures by the bronchial explant culture technique that we use in our studies. There is no doubt that further research to enable the use of bronchial explants after multiple outgrowths to obtain BEC cultures over longer time periods will be of great advantage, provided that the structural and biochemical characteristics of these cultures are not affected by such a procedure.

The explant cell culture technique has been successfully used by investigators for obtaining BEC cultures (22–24). This method allows us to obtain BEC cultures with high vitality and purity. However, the main problem in obtaining primary BEC cultures is the difficulty in recruiting adequate numbers of suitable and eager donors. Therefore, it is critical for the researcher to use donor explants to a maximum. Alternatively, the limited number of bronchial explants adhering to culture plates for generating cells poses a serious disadvantage for the researcher, as we observed in our current studies. Our findings suggest that this limitation may be associated with decreased P/S cell numbers in both bronchial explants and BEC cultures. Therefore, it is possible that strategies aimed toward keeping P/S cell numbers in both explants and BEC cultures at steady-state levels would enable the researcher to obtain successful BEC cultures during advancing outgrowth numbers.

Another problem that may be encountered in primary culture studies, is that BECs cultured from advanced outgrowths may exhibit biochemical and physiologic properties differently to cells outgrown from the explant at first use. Indeed, in our previous studies, although the basal inflammatory mediator release from atopic asthmatic BEC cultures obtained at the first use of explants was markedly higher than the release from cells of non-atopic and non-asthmatic individuals, the levels of these mediators were not different in both cell groups obtained from the second outgrowth of the explant (36). Interestingly, following exposure to pollutants such as ozone and NO_2_, the release from asthmatic cells was significantly increased compared to cells of non-asthmatic subjects. Whether such a phenomenon is due to changes in P/S cell expression remains to be determined. Alternatively, it could be related to changes in subepithelial extracellular matrix (ECM), as it has been shown that bronchial ECM from COPD patients induces differential gene expression in primary normal human bronchial epithelial cells compared to normal bronchial ECM (37).

Furthermore, we cultured BEC cultures on submerged coverslips, intact solid surfaces, which may have a direct effect upon the stemness/progenitor cell population, instead of alternative methods such as air–liquid interface cultures, known to better differentiate and preserve the phenotypic characteristics of epithelial cells (36, 38). However, we needed to use cultures established on coverslips for immune staining process of P/S markers. Indeed, we still observed differences in expression of the P/S, and cell numbers outgrowing from explants, when transferring explants for establishing new cultures. Additionally, there were differences between BEC cultures from smokers without COPD and COPDs in the expression of P/S markers, suggesting that cells still retain their phenotypic characteristics.

A major limitation to our current study was that we were not able to establish a control group of non-smokers without COPD. Unfortunately, almost all the donors, whose tissues were used for this study, were smokers with or without COPD. Studies that can be conducted in non-smokers will help us to understand the effects of cigarette smoking on P/S cell expression in both airway epithelium and BEC cultures. The fact that the majority of the patients included in the study were men, may be related to the higher smoking rates in males (41.7%) than in females (14.1%) in Turkey. As a result, the number of male patients underwent surgery because of lung cancer, which is mainly associated with cigarette smoking, was much higher than that of females (39).

## Conclusion

In summary, our findings have demonstrated that P/S cell markers are decreased in both bronchial explants and BEC cultures as outgrowth number increases, and that this may be more pronounced in tissue from COPD patients. Furthermore, the adherence of bronchial explants to culture plates and their capacity in generating BECs were decreased with advanced outgrowth number. Taken together, our findings suggest that the number of P/S cells present in the airway epithelium may play a role in the pathogenesis of COPD, and that the amount of these cells is of importance for obtaining primary airway epithelial cell cultures *in vitro*.

## Data availability statement

The original contributions presented in the study are included in the article/Supplementary material, further inquiries can be directed to the corresponding author.

## Ethics statement

The studies involving humans were approved by The Ethics Committee of Gaziantep University (Ref no: 01/2011–08). The studies were conducted in accordance with the local legislation and institutional requirements. Written informed consent for participation in this study was provided by the participants’ legal guardians/next of kin.

## Author contributions

NB, HB, and AE: conceptualization. SK, NB, OK, NK, HR, and HB: data curation. SK, NB, OK, NK, HR, AY, and HB: formal analysis. HB: funding acquisition and project administration. NB, KB, OK, NK, and HB: investigation and methodology. KB and HB: resources. AE and HB: supervision. KB and MY: validation. NB and SK: writing – original draft. NB, HB, OK, NK, HR, AY: writing – review and editing. All authors contributed to the article and approved the submitted version.

## Supplementary material

SUPPLEMENTARY FIGURE S1 Correlation between age, cigarette smoke, lung function parameters (FEV1%, FVC%, and FEV/FVC) and expression of CK5, CK14 and p63 in bronchial epithelial explants from smokers without COPD (smokers) and smokers with COPD (COPD). Correlation coefficients (*r*) and *p* values are presented in the table below the figure.

SUPPLEMENTARY FIGURE S2 Correlation (“*r*”) between age, cigarette smoke, lung function parameters (FEV1%, FVC%, and FEV/FVC) and expression of CK5, CK14 and p63 in bronchial epithelial cell cultures from smokers without COPD (smokers) and smokers with COPD (COPD). Correlation coefficients (*r*) and *p* values are presented in the table below the figure.

SUPPLEMENTARY FIGURE S3 Correlation (“*r*”) between age, cigarette smoke (pack/years), lung function parameters (FEV1%, FVC%, and FEV/FVC) and number of explants generating cells, total epithelial cell counts, and cells/explants from smokers without COPD (smokers) and smokers with COPD (COPD). Correlation coefficients (*r*) and *p* values are presented in the table below the figure.
