# Supplementary figures and images for: The effect of multiple outgrowths from bronchial tissue explants on progenitor/stem cell number in primary bronchial epithelial cell cultures from smokers and patients with COPD
Source: Front Med (Lausanne). 2023 Oct 13;10:1118715. doi: 10.3389/fmed.2023.1118715 (PMC10614425; doi:10.3389/fmed.2023.1118715)

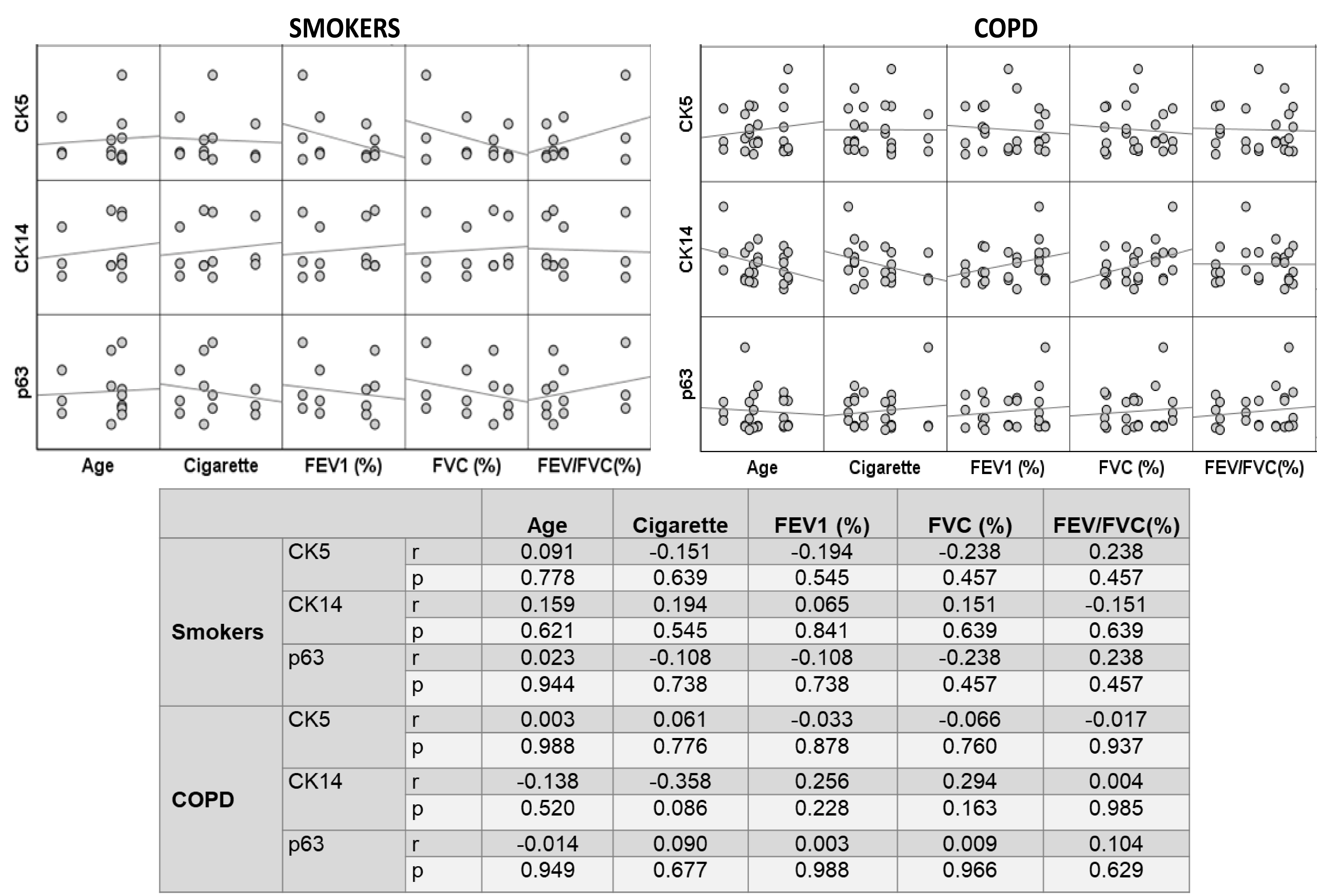

Supplement: SUPPLEMENTARY FIGURE S1 — Correlation between age, cigarette smoke, lung function parameters (FEV1%, FVC%, and FEV/FVC) and expression of CK5, CK14 and p63 in bronchial epithelial explants from smokers without COPD (smokers) and smokers with COPD (COPD). Correlation coefficients (r) and p values are presented in the table below the figure. [file Image_1.JPEG]

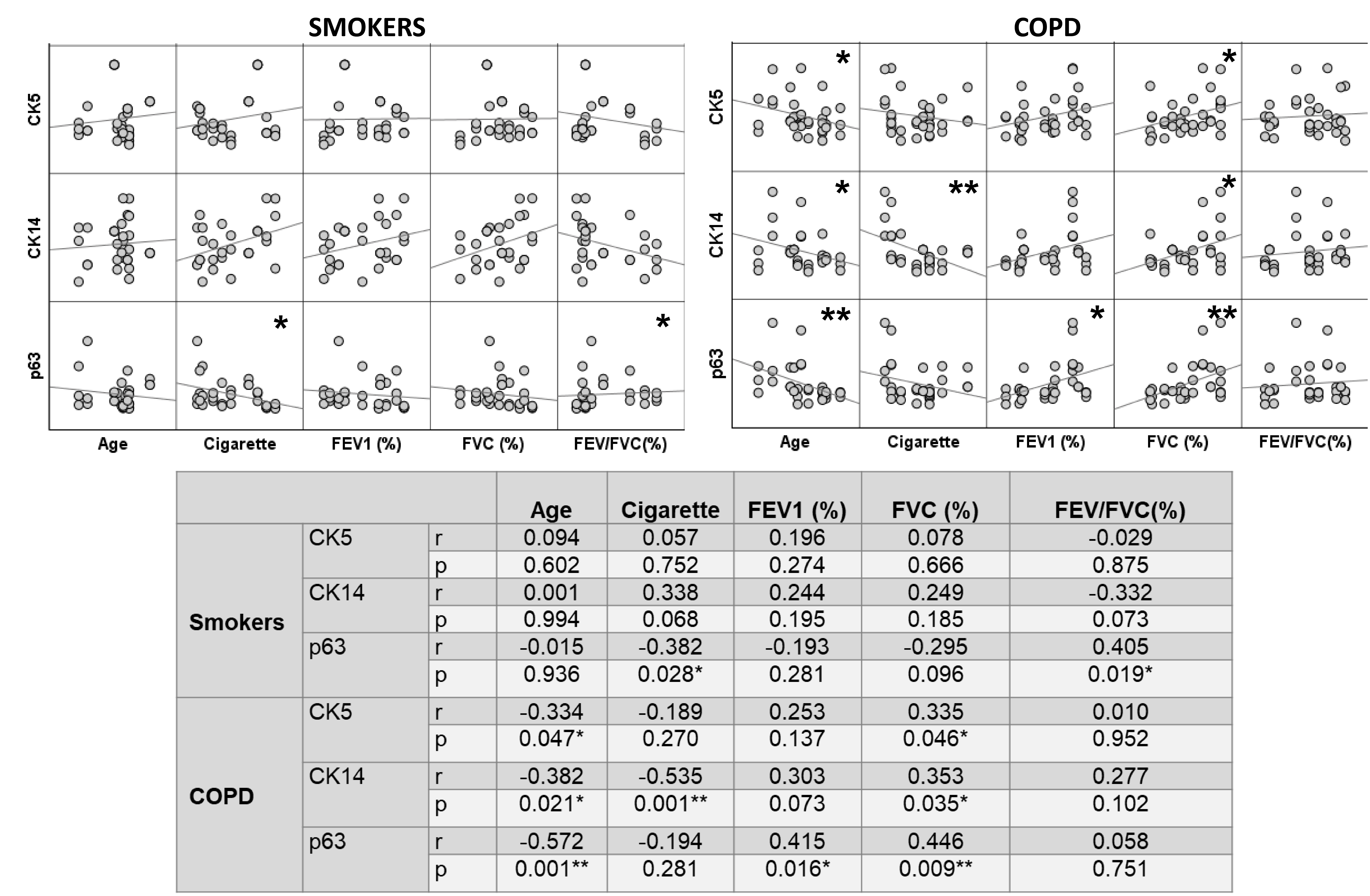

Supplement: SUPPLEMENTARY FIGURE S2 — Correlation (“r”) between age, cigarette smoke, lung function parameters (FEV1%, FVC%, and FEV/FVC) and expression of CK5, CK14 and p63 in bronchial epithelial cell cultures from smokers without COPD (smokers) and smokers with COPD (COPD). Correlation coefficients (r) and p values are presented in the table below the figure. [file Image_2.JPEG]

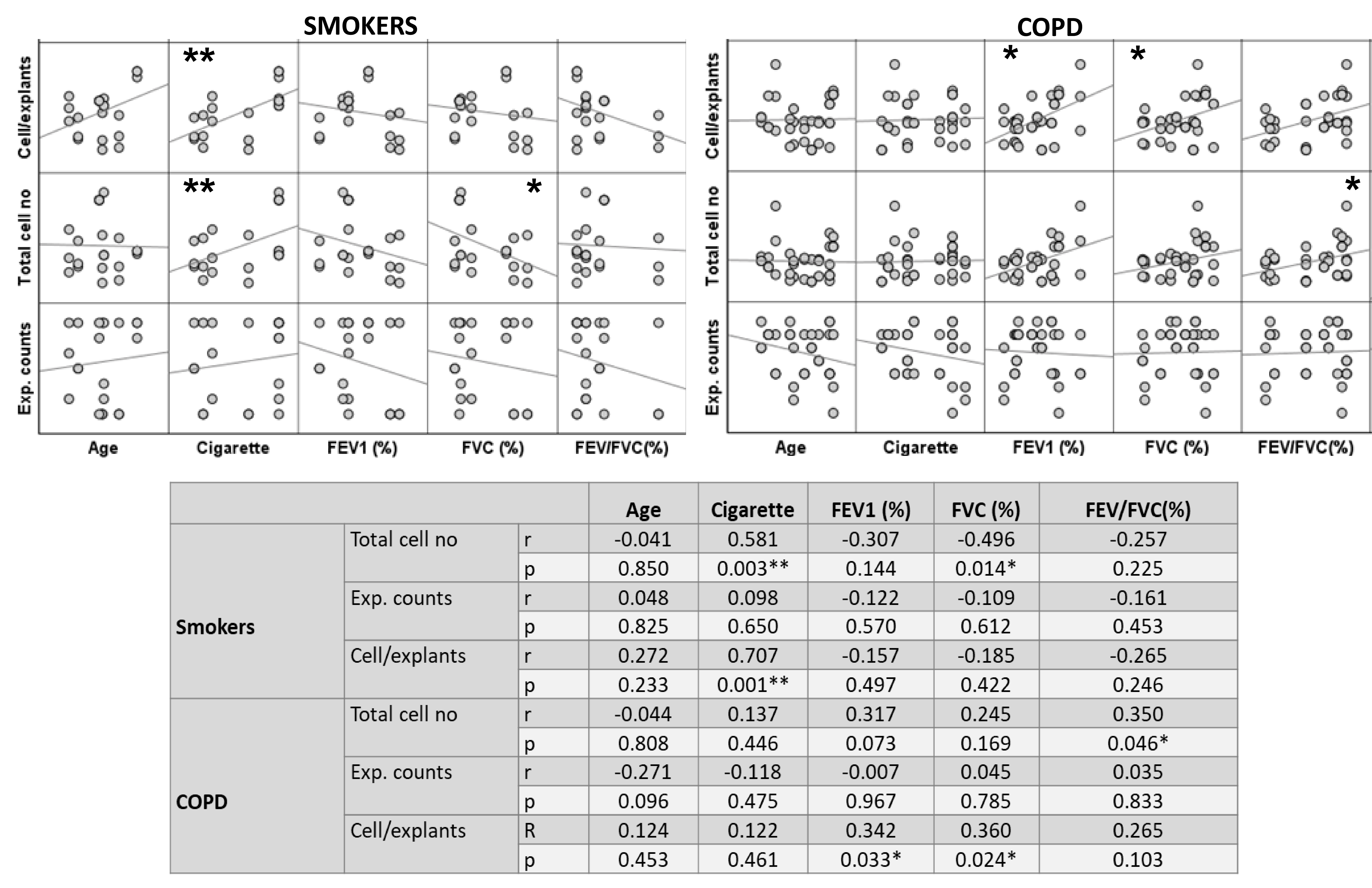

Supplement: SUPPLEMENTARY FIGURE S3 — Correlation (“r”) between age, cigarette smoke (pack/years), lung function parameters (FEV1%, FVC%, and FEV/FVC) and number of explants generating cells, total epithelial cell counts, and cells/explants from smokers without COPD (smokers) and smokers with COPD (COPD). Correlation coefficients (r) and p values are presented in the table below the figure. [file Image_3.JPEG]
