# Supplementary material for: The effect of multiple outgrowths from bronchial tissue explants on progenitor/stem cell number in primary bronchial epithelial cell cultures from smokers and patients with COPD
Source: Front Med (Lausanne). 2023 Oct 13;10:1118715. doi: 10.3389/fmed.2023.1118715 (PMC10614425; doi:10.3389/fmed.2023.1118715)
Supplement: Supplementary file 4 [file Table_1.docx]

Supplementary Material

**The effect of multiple outgrowths from bronchial tissue explants on progenitor/stem cell number in primary bronchial epithelial cell cultures from smokers and patients with COPD**

**Nuray Bostancieri^1,2^, Kemal Bakir^3,#a^, Seval Kul^4^, Ayhan Eralp^1,#b^, Ozgecan Kayalar^5^, Nur Konyalilar^5^, Hadi Rajabi^5^, Mehmet Yuncu^1^, Ali Oender Yildirim^5,6^, Hasan Bayram^2,5*^**

|  |
| --- |

^1^Department of Histology and Embryology, School of Medicine, University of Gaziantep, Gaziantep, Turkey.

^2^Cell Culture Laboratory, Department of Chest Diseases, School of Medicine, University of Gaziantep, Gaziantep, Turkey.

^3^Department of Pathology, School of Medicine, University of Gaziantep, Gaziantep, Turkey.

^4^Department of Biostatistics, School of Medicine, University of Gaziantep, Gaziantep, Turkey.

^5^Koc University Research Center for Translational Medicine, Koc University, Istanbul, Turkey.

^6^Comprehensive Pneumology Center (CPC), Institute of Lung Biology and Disease, Helmholtz Zentrum München, 85764 Munich, Germany.

^#a^Current Address: Department of Pathology, School of Medicine, Sanko University, Gaziantep, Turkey.

^#b^Current Address: Department of Histology and Embryology, Faculty of Medicine Namik Kemal University, Tekirdag, Turkey.

***Corresponding Author:**

**Hasan Bayram MD, PhD.**

Koc University Hospital, Department of Pulmonary Medicine, Maltepe Mah. Davutpasa Cad. No. 4, Zeytinburnu, Istanbul 34010, Turkey.

Tel: +90 850 250 8 250 x29993

E-mail: [habayram@ku.edu.tr](mailto:habayram@ku.edu.tr)

**SUPPLEMENTARY TABLES:**

**S1 Table.** Bronchial epithelial cells expressing progenitor/stem cell markers in explants obtained from smokers and patients with COPD, and effects of outgrowth number.

|  | Tr1 | Tr2 | Tr3 |
| --- | --- | --- | --- |
| **Smoker** |  |  |  |
| CK5 | 39.50 (29.48-51.80) | 16.65 (15.28-19.43) | 12.80 (10.40-14.73)*** |
| CK14 | 52.80 (45.10-57.63) | 28.15 (26.25-29.95) | 22.60 (19.38-25.65)*** |
| P63 | 39.30 (30.58-44.40) | 22.90 (18.50-25.45) | 16.50 (13.08-18.23)*** |
| **COPD** |  |  |  |
| CK5 | 46.95(44.40-65.88) | 18.15(13.78-25.10) | 9.20(7.85-15.25)** |
| CK 14 | 32.50(27.50-36.75)^†^ | 17.60(12.93-19.75)^†^ | 10.55(8.15-12.38)***^,^ ^†^ |
| P63 | 35.80(32.38-47.20) | 19.60(14.50-27.55) | 13.30(12.23-13.98)*** |

Tr, tissue transfer/outgrowth number; COPD, chronic obstructive pulmonary disease; CK**,** cytokeratin. *p<0.01, **p<0.001, and ***p<0.0001 vs Tr 1; ^†^p=0.001 vs smokers.

**S2 Table.** Bronchial epithelial cells expressing progenitor/stem cell markers in cultures btained from smokers and patients with COPD, and the effect of outgrowth number.

|  | Tr1 | Tr2 | Tr3 |
| --- | --- | --- | --- |
| **Smokers** |  |  |  |
| CK5 | 98.90 (78.80- 137.8) | 53.60 (37.60- 66.80)** | 58.50(44.13- 70.93)* |
| CK14 | 100.2± 7.70 | 59.87± 6.22** | 74.20± 9.28 |
| P63 | 103.2± 20.49 | 53.16± 20.49* | 47.32± 7.34* |
| **COPD** |  |  |  |
| CK5 | 47.47± 4.57^†††^ | 30.93± 3.26*^,††^ | 26.13± 2.64**^,††^ |
| CK 14 | 39.45(27.05- 49.03) ^†††^ | 29.60(22.15- 32.85) ^†††^ | 19.40(13.10- 27.00)**^,†††^ |
| P63 | 26.30(21.50- 55.00)^†^ | 23.10(17.15- 27.75) ^††^ | 27.75(6.60- 21.10)**^,†^ |

**Tr**, tissue transfer/outgrowth number; **COPD**, chronic obstructive pulmonary disease; **CK,** cytokeratin. *p<0.01, **p<0.001, and ***p<0.0001 vs Tr 1; ^†^p<0.05, ^††^p<0.01, and ^†††^p<0.001 vs smokers.

**S3 Table.** Effect of outgrowth number on the number of explants adhering to culture dishes and generating cells, total cell counts and cell/explant obtained from smokers and patients with COPD.

|  | **Tr1** | **Tr2** | **Tr3** |
| --- | --- | --- | --- |
| **Smokers**  **Number of explants** | 6±0.0 | 3.3±0.68** | 1.6±0.71** |
| **Total cell counts** | 57027 (49219-76806) | 28125 (17187-33593)** | 15625(3711-21094)** |
| **Cell number/Explant** | 10677 (8463-14444) | 6718 (2864-10416) | 3698 (1497-10313) |
| **COPD** |  |  |  |
| **Number of explants** | 6.1±0.16 | 4±0.48****** | 2.8±0.63** |
| **Total cell counts** | 56250 (50000-87500) | 14453 (3906-59375) * | 28906 (14453-46875)* |
| **Cell number/Explant** | 8631(8177-14583) | 2435 (1302-10938) | 7031 (6120-12044) |

**Tr**, tissue transfer/outgrowth number; **COPD**, chronic obstructive pulmonary disease. *p<0.05, **p<0.0001 vs Tr 1.
